# PI3 K/AKT/mTOR pathway and its role in breast cancer stem cells

**DOI:** 10.1007/s00210-025-04297-3

**Published:** 2025-07-17

**Authors:** Kirti S. Prabhu, Shilpa Kuttikrishnan, Zahwa Mariyam, Ummu Habeeba, Anu Jayanthi Panicker, Tariq Masoodi, Kulsoom Junejo, Shahab Uddin

**Affiliations:** 1 Translational Research Institute, Academic Health System, Hamad Medical Corporation, PO Box 3050, Doha, 3050 Qatar; 2 General Surgery Department, Hamad General Hospital, Hamad Medical Corporation, Doha, 3050 Qatar; 3 Dermatology Institute, Academic Health System, Hamad Medical Corporation, Doha, 3050 Qatar; 4 Laboratory of Animal Research Center, Qatar University, Doha, 2713 Qatar; 5 Department of Biosciences, Integral University, Lucknow, Uttar Pradesh 226026 India; 6 Sidra Medicine, Doha, Qatar

**Keywords:** Breast cancer, Cancer stem cells, Epithelial–mesenchymal transition, PI3/AKT/mTOR

## Abstract

Cancer stem cells (CSCs) are a small subpopulation bearing self-renewal ability, mediating tumor initiation and propagation. Several molecular pathways, including the PI3K/AKT/mTOR pathway, are known to be aberrantly activated in cancers. In CSCs, PI3K/AKT/mTOR pathway has been associated with attribution of various properties to cancer cells including stemness characteristics, proliferation, migration, epithelial to mesenchymal transition, and autophagy. Thus, targeting PI3K/AKT/mTOR pathway with novel inhibitors might help to control the growth and proliferation of the breast CSC population. Though many studies have focused on PI3K/AKT/mTOR pathway in breast cancer, limited literature is available on the role of PI3K/AKT/mTOR pathway in breast CSCs. Here, in our present review, we have highlighted the role of the PI3K/AKT/mTOR signaling pathway in breast CSCs and its applications in therapeutic targeting.

## Introduction

The Global Cancer Observatory (GLOBOCAN) reports that breast cancer (BC) ranks among the most prevalent cancers worldwide (Sung et al. 2021). The 5-year survival rate of BC exhibits considerable variation based on geographic region and stage of diagnosis. In high-income nations, the 5-year survival rate for BC frequently exceeds 90% owing to early diagnosis and sophisticated treatments; however, in low-resource countries, survival rates may be significantly poor (Sung et al. 2021; Katsura et al. 2022). Several significant risk factors including family history (Brewer et al. 2017), age (McGuire et al. 2015), race (Hill et al. 2019; Yedjou et al. 2019), reproductive status (Albrektsen et al. 2005), use of certain drugs (Vinogradova et al. 2020; Narod 2011), Body Mass Index (Wang et al. 2019), physical activity (Chen et al. 2019), alcohol consumption (Erol et al. 2019; Mostofsky et al. 2025), smoking (Jones et al. 2017), exposure to artificial light (Johns et al. 2018), and chemical exposure (Eve et al. 2020) perform a key function in the development and progression of BC.

Based on HER2 amplification, hormone receptor status, and genomic profiling, BC is molecularly divided into many subtypes that influence prognosis and therapeutic approaches. The four main subtypes are luminal A (ER/PR-positive, HER2-negative, low Ki67), luminal B (ER-positive, HER2-negative/positive with increased proliferation), HER2-enriched (HER2-positive, ER/PR-negative), and triple-negative/basal-like (ER/PR/HER2-negative) (Kinsella et al. 2013; Tsang et al. 2020). Luminal B breast cancer exhibits sensitivity to endocrine therapy; yet, certain patients may experience primary or secondary resistance to such treatment in contrast to Luminal A, which exhibits favorable responses to endocrine therapy and possesses a favorable prognosis (Yang et al. 2022; Parise et al. 2009; Park et al. 2012; DePolo J 2024). Anti-HER2 treatments improve survival for HER2-enriched subtypes (12–20%) even though their prognosis has previously been poor. Although new immunotherapies show promise, triple-negative tumors (15–20%) are aggressive and lack targeted therapeutics. They are frequently associated with BRCA1 mutations. Sensitive markers to predict chemotherapy sensitivity and whether patients can benefit from particular chemotherapy regimens are lacking (Orrantia-Borunda et al. 2022).

Breast cancer management often encompasses a combination of surgical intervention, radiation, chemotherapy, hormone therapy, and targeted therapies (Harbeck and Gnant 2017; Nicolini et al. 2018). Despite these thorough strategies, recurrence and treatment resistance persist as considerable obstacles, especially in specific subtypes (Harbeck and Gnant 2017). There is mounting evidence that these issues are largely caused by cancer stem cells (CSCs). CSCs represent a limited population of tumor cells capable of self-renewal and evading standard therapies through mechanisms including drug efflux, augmented DNA repair, and dormancy. Moreover, CSCs engage with the tumor environment to facilitate immune evasion and maintain their stem-like characteristics (Chu et al. 2024; Pan et al. 2025; Beziaud et al. 2023; Yamashina et al. 2014).

## Breast cancer stem cells

**Fig. 1:**
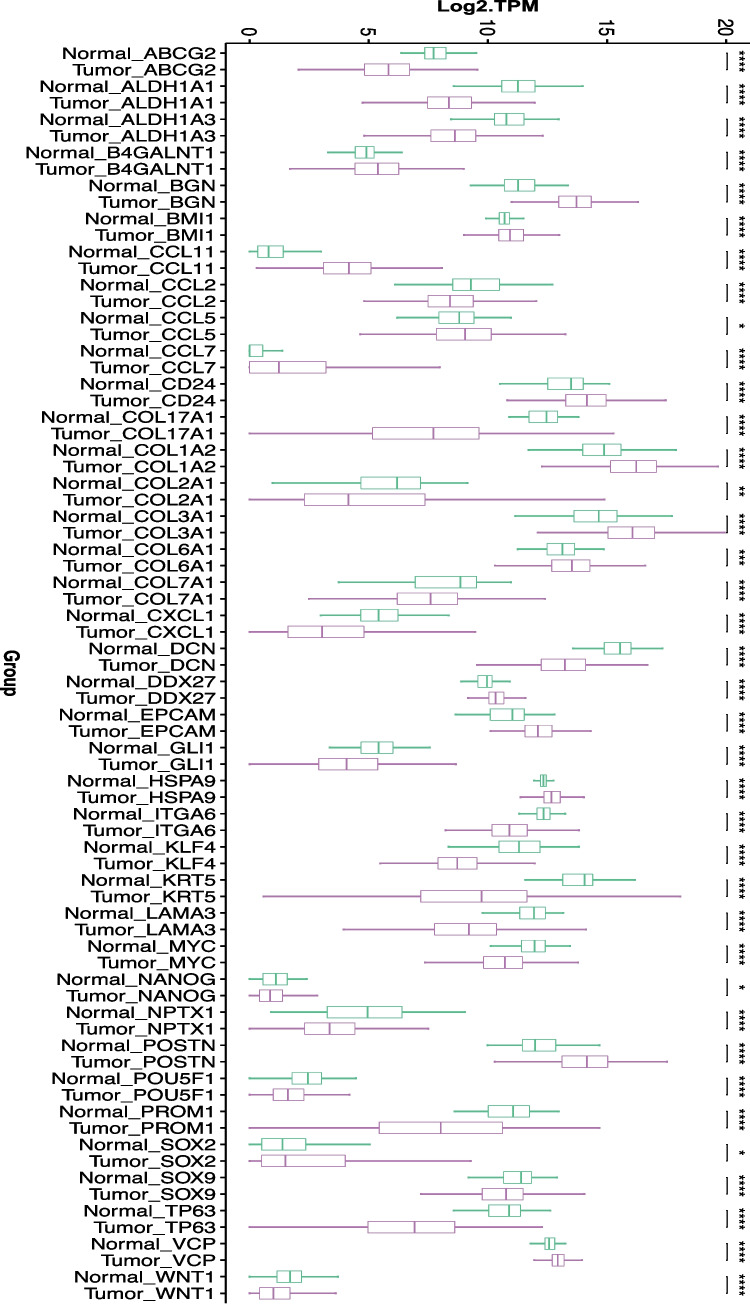
Approximately 180 breast cancer stem cell (CSC) gene markers were retrieved from InnateDB (Breuer et al. 2013) along with confidence scores. Normalized expression count data of 49 high confidence CSC genes (confidence score > 0.3) was retrieved from TCGA breast cancer and normal tissues using UCSC Xena server (https://xena.ucsc.edu). Differential expressions between 114 normal and 1097 cancer tissues were calculated using Mann–Whitney *U* test and the statistically significant genes were plotted using boxplots

In BC, CSCs were first identified and isolated by Al-Hajj et al. leading to the identification of specific CSC markers, CD44^+^/CD24^−^/EpCAM (Al-Hajj et al. 2003). Based on these markers, the authors showed that CSCs were able to induce tumor in mice indicating the role of CSC in tumor initiation and progression (Loh and Ma 2024; Ozsvari et al. 2017). BCSCs are a rare, aggressive tumor subpopulation with self-renewal and differentiation capabilities, driving metastasis and therapy resistance (Jan et al. 2025; Abreu de Oliveira et al. 2021). There are several markers specific to CSCs, including CD133, aldehyde dehydrogenase (ALDH), and SRY-box transcription factor 2 (Sox2); octamer-binding transcription factor 4 (Oct4) and Nanog have also been identified to play a significant role in its stemness characteristics (Chu et al. 2024). With regards to their role in drug resistance, CSCs are well characterized to express ATP binding cassette (ABC) drug transporters, including the ATP-binding cassette subfamily G member 2 (ABCG2) that facilitates efflux of therapeutic drugs against the concentration gradient, thereby initiating drug resistance pathways (Damiani and Tiribelli 2024; Robey et al. 2001). Compared to normal tissue samples, CSCs showed a significant upregulation of around 180 genes (Fig. 1). On the other hand, CSCs are also known to modulate several signaling pathways, including Wingless/Integrated (Wnt)/beta-catenin (β-Catenin) (Zhao et al. 2024; Lv et al. 2015), Phosphatidylinositol 3-kinase/protein kinase B (PI3 K/AKT/MTOR) (Karami Fath et al. 2022; He et al. 2019), NOTCH, Sonic Hedgehog (Berrino and Omar 2024), and Nuclear factor kappa B (NF-κB) (Guo et al. 2024; Chen et al. 2011) pathways for their growth, self-renewal, and metastatic capabilities (Yi et al. 2025).

The aberrant PI3 K/AKT/mTOR pathway is known to be involved in cancer (Garg et al. 2025; Fanucci et al. 2024). However, limited studies on the role of CSCs in modulating PI3 K/AKT/mTOR pathways for BC progression and chemoresistance have been documented (Garg et al. 2025). Since this pathway is considered critical in cancer progression, we aim to focus this review on the role of PI3 K/AKT/mTOR signaling pathways in growth, survival, maintenance of BCSCs, and their role in therapeutic resistance. The review further aims to shed light on BCSCs as potential candidates for therapeutic targeting.

## Structure, functions, and role of PI3 K/AKT/mTOR pathway

The PI3 K/AKT/mTOR pathway is an essential intracellular signaling cascade that regulates cell growth, survival, metabolism, and proliferation. The structure consists of three primary components: PI3 K (phosphoinositide 3-kinase), a heterodimeric enzyme made up of regulatory (p85) and catalytic (p110) subunits. The active PI3 K phosphorylates PIP2 to become PIP3, which subsequently activates AKT, a serine/threonine kinase with a pleckstrin homology (PH) domain that binds to PIP3 for membrane localization. The mTOR (mammalian target of rapamycin) consists of two complexes (mTORC1 and mTORC2) that are involved in nutrition and growth signaling. The activation of PI3 K by receptor tyrosine kinases (RTKs) or GPCRs triggers AKT phosphorylation, which then affects downstream effectors like GSK-3β, FOXOs, and TSC1/2. mTORC1 promotes protein synthesis via S6 K and 4EBP1, while mTORC2 regulates cytoskeletal dynamics and AKT activation. This system is essential for physiological activities like as insulin signaling and tissue homeostasis; yet, its dysregulation—caused by mutations (e.g., PIK3 CA, PTEN loss) or overactivation—leads to cancer (Glaviano et al. 2023; Asati et al. 2016).

**Fig. 2:**
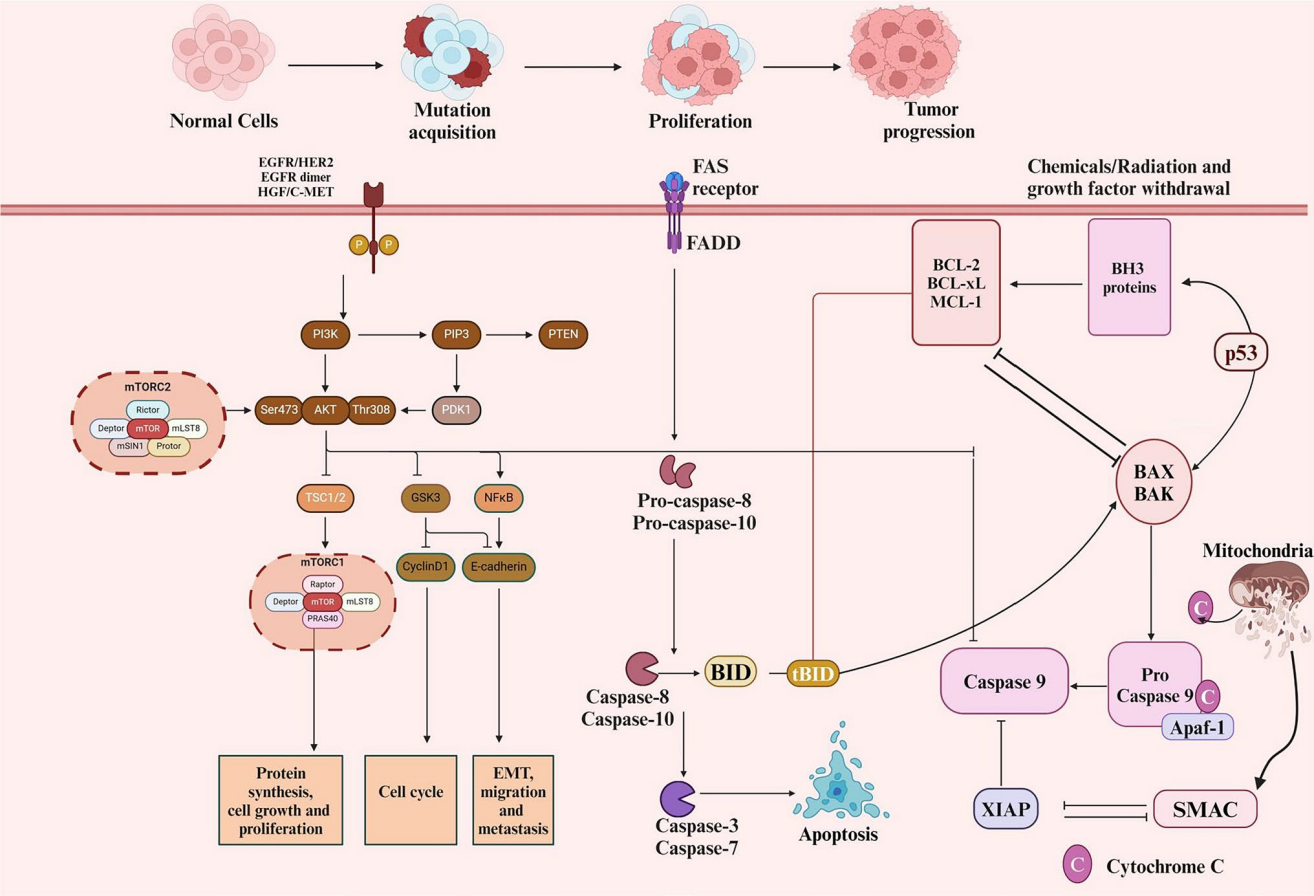
Interaction of mTOR with other signaling pathways. The phosphoinositide 3-kinase (PI3 K)/mTOR pathways react to external and intracellular signals and extensively interact to modulate one another. Growth factors bind to receptor tyrosine kinases (RTK), activating the PI3 K pathway through the regulation of a phosphorylation cascade. Activated PI3 K phosphorylates PIP2 to produce membrane-associated PIP3, subsequently activating AKT. The activation of mTORC1 and mTORC2 governs cell survival, proliferation, angiogenesis, and other related functions

The PI3 K/AKT/mTOR signaling pathways are highly conservative intracellular signal transduction pathways crucial for cell growth, differentiation, apoptosis, angiogenesis, and survival (Kilmister and Tan 2025; Mousavikia et al. 2025; Li et al. 2020; Mortazavi et al. 2022; Li et al. 2022; Tariq and Luikart 2021; Dworakowska et al. 2009). Figure 2 showcases crosstalk between mTOR and other signaling pathways.

### Receptor tyrosine kinases

Receptor tyrosine kinases (RTKs) are a subclass of tyrosine kinases that are high-affinity cell surface receptors for several growth factors, cytokines, and hormones and are involved in mediating cell-to-cell communications (Tomuleasa et al. 2024). This receptor has identified three functional domains: an extracellular domain that binds with the ligand, a transmembrane domain spanning the plasma membrane, and an intracellular region containing a tyrosine kinase domain (TKD) and a carboxyl-terminal tail (Trenker and Jura 2020). Upon ligand binding and activation, two RTK molecules form a dimer, activating the intracellular TKD leading to autophosphorylation of these monomers (Tomuleasa et al. 2024). Autophosphorylation of TKDs foster various proteins with Src homology-2 or phosphotyrosine-binding (PTB) domains. These proteins further mediate several critical signaling pathways by interacting with different molecules controlling several cellular responses (Diop et al. 2022; Kim et al. 2023).

### Phosphatidylinositol-3-kinase

The phosphatidylinositol 3-kinase (PI3 K) is a plasma membrane–associated heterodimer composed of the regulatory p85 and catalytic p110 subunits, which work together to mediate RTK-dependent signaling. The p85 regulatory subunit stabilizes the p110 catalytic subunit while maintaining basal inhibition, and activation occurs when phosphorylated motifs interact with active RTKs or adaptor proteins. This enzyme phosphorylates PIP2 into PIP3 and introduces AKT and 3-phosphoinositide-dependent kinase 1 (PDK1) into the plasma membrane. This spatial rearrangement enables PDK1 to phosphorylate and activate AKT, initiating downstream signaling cascades that control cell survival, growth, and metabolic processes (Li et al. 2024) (Garcia-Viloca et al. 2022; Kearney et al. 2021; Gozzelino et. al. 2020).

### Protein kinase B

Protein kinase B (AKT) family of proteins includes kinases specific to serine/threonine residues of target proteins (Han et al. 2024). Three isoforms of AKT have been identified: AKT1, expressed in most of the tissues; AKT2, expressed mainly in insulin-sensitive tissues; and AKT3, expressed in the brain and testicles (Kumar et al. 2025; Adon et al. 2025). Upon their recruitment to the cell membrane by PIP3, AKT is phosphorylated partially by mammalian target of rapamycin complex 2 (mTORC2) on Ser473 in the carboxy-terminal hydrophobic motif, which imparts conformational changes to AKT (Jhanwar-Uniyal et al. 2019). This further facilitates the phosphorylation at Thr308 by PDK1, thereby accomplishing AKT activation (Zheng et al. 2023) leading to cell adhesion, proliferation, survival, and activation of its downstream targets (Singh et al. 2025; Toson et al. 2022).

### Mammalian target of rapamycin

Mammalian target of rapamycin (mTOR), also referred to as the mechanistic target of rapamycin, is a large serine/threonine kinase that is constitutively expressed in mammalian cells (Qiang et al. 2025). There are two mTOR complexes, mTORC1 and mTORC2, found in mammalian cells (Qiang et al. 2025). Structurally, mTORC1 contains mTOR, Raptor, and Mammalian Lethal with SEC13 protein 8 (mSLT8) that enhance the kinase activity (Qiang et al. 2025; Hua et al. 2019). Through the downstream effectors, 4EBP1 and P70S6 kinase (S6 K), it initiates the translation of mRNA into proteins promoting cell growth and metabolism. The role of mTORC1 in de novo lipid synthesis is also reported (Wu et al. 2022). mTORC2 complex encompasses six proteins, namely, DEPTOR, SIN1, RICTOR, mLST8, and mTOR, and is majorly involved in phosphorylating and activating AKT, thereby promoting cell proliferation and survival (Unni and Arteaga 2019; Ragupathi et al. 2024). Studies have also shown the role of mTOR in several other cell fate–determining events, such as apoptosis and autophagy (Khayatan 2025; Zeng et al. 2024).

### Phosphoinositide-dependent kinase 1

Phosphoinositide-dependent kinase 1 (PDK1), a 63-kDa serine-threonine kinase, functions as a key regulator of the AGC kinase superfamily (PKA, PKG, PKC), predominantly activating AKT via phosphorylation at Thr308. The pleckstrin homology (PH) domain binds PIP2 and PIP3, facilitating membrane localization and substrate activation (Mousavikia et al. 2025; Xiang et al. 2025; Wang et al. 2022; Levina et al. 2022; Sacerdoti et al. 2023). Overexpression of PDK1 accelerates tumor growth in several malignancies, with continuing activation of the PI3 K/PDK1/AKT pathway (Tao et al. 2024; Glaviano et al. 2023; Agrawal et al. 2025; Hao et al. 2025; Watt and Goel 2022; Ippen et al. 2019). In breast cancer (BC), PDK1 is genomically amplified and overexpressed, correlating with advanced tumor stage and poor prognosis. In comparison to benign lesions, BC demonstrates elevated phosphorylation at Ser-241, indicating PDK1 activation. Functional studies demonstrate PDK1’s critical role in BC formation and metastasis by facilitating cell proliferation, survival, and glycolytic reprogramming. In BC, PDK1 is genomically amplified and overexpressed, correlating with advanced tumor stage and poor prognosis 19,602,588 (Wang et al. 2022). BC shows increased phosphorylation at Ser-241, a marker of PDK1 activation, in comparison to benign lesions (Levina et al. 2022; Wang et al. 2022). In addition to oncogenic signaling, PDK1 engages with cyclin-dependent kinase 1 (Cdk1) to modulate stem cell self-renewal and pluripotency during cellular reprogramming. This interaction indicates PDK1’s dual function in sustaining malignant and stem-like characteristics. In BCSC-enriched tumors, therapeutic targeting of PDK1 holds promise for overcoming resistance, especially when combined with PI3 K/AKT inhibitors (Wang et al. 2017; Varzideh et al. 2023; Martin 1981).

### Phosphatase and tensin homolog

As a negative regulator of the PI3 K/AKT/mTOR signaling pathway, phosphatase and tensin homolog (PTEN) catalyzes the dephosphorylation of PIP3 to PIP2 (Luongo et al. 2019; Maehama and Dixon 1998; Liu et al. 2024). One tumor suppressor gene that is often changed in malignancies is PTEN, and it controls the process by which stem cells regenerate themselves (Abdelaziz et al. 2023; Zhang et al. 2018). Several studies have shown that PTEN has a crucial impact on normal stem cells as well as CSC homeostasis wherein loss of PTEN has been reported to promote the growth and survival of CSCs (Korkaya et al. 2009; Al-Dhfyan et al. 2017). It has been shown that PTEN inhibits epithelial–mesenchymal transition (EMT) and cancer stem cell activity by reducing the expression of Abi1 (Qi et al. 2020). Overexpression of Abi1 in non-tumorigenic mammary epithelial cells leads to the induction of the EMT and an increase in the activity of stem/progenitor cells. However, when Abi1 is depleted in BC, it hinders the process of EMT and reduces the activity of CSCs, which is like the effect of reintroducing PTEN (Qi et al. 2020). PTEN suppression results in the accumulation of both normal and malignant mammary stem cells and induces a substantial elevation in the levels of AKT phosphorylation. This, in turn, leads to the activation of β-catenin through GSK3β-dependent mechanisms, which promotes the progression of malignant transformation. Consistently, the administration of perifosine (an inhibitor of AKT) or Ly294002 (an inhibitor of PI3 K), either alone or in conjunction with chemotherapy, decreases the population of mammary stem cells and inhibits the formation of tumors in BC xenografts (Korkaya et al. 2009; Wylaz et al. 2023).

### PI3 K/AKT/mTOR mediated regulation of cancer stem cell markers

**Fig. 3:**
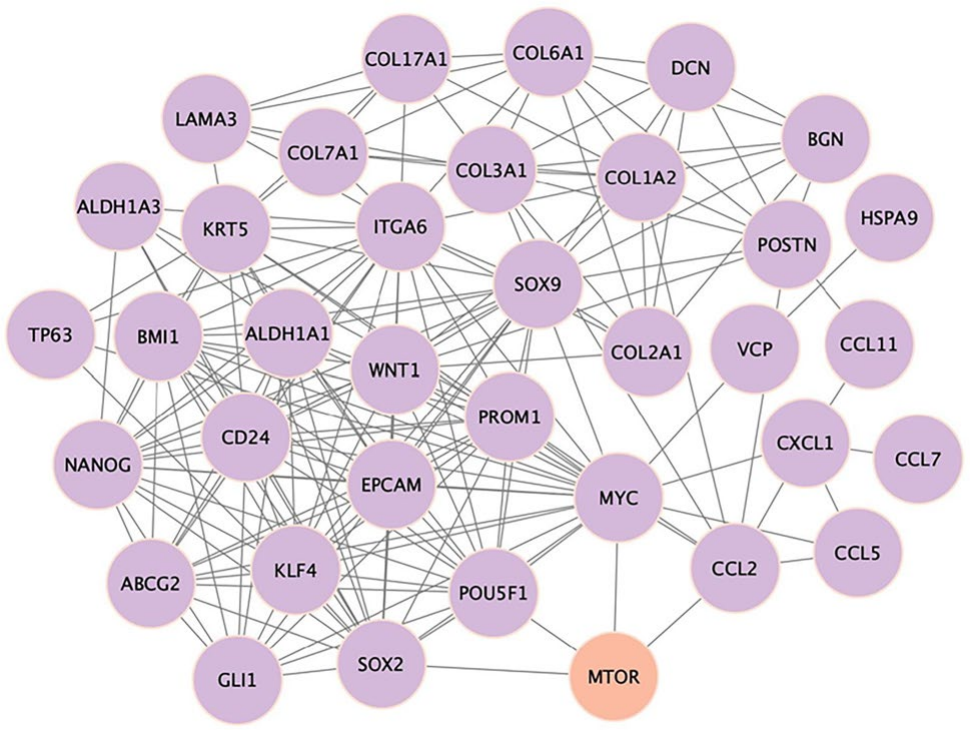
A gene regulatory network, showing interactions between MTOR with CSC genes. Network analysis of high confidence dysregulated CSC genes analyzed using STRING and visualized using Cytoscape. The network shows direct and indirect connections of CSC genes with *MTOR*. Each node represents a gene, and the lines between them indicate regulatory relationships or interactions. This network aids in illustrating the complex interplay between these genes and can be utilized to explore implications in disease mechanisms or therapeutic strategies

**Fig. 4:**
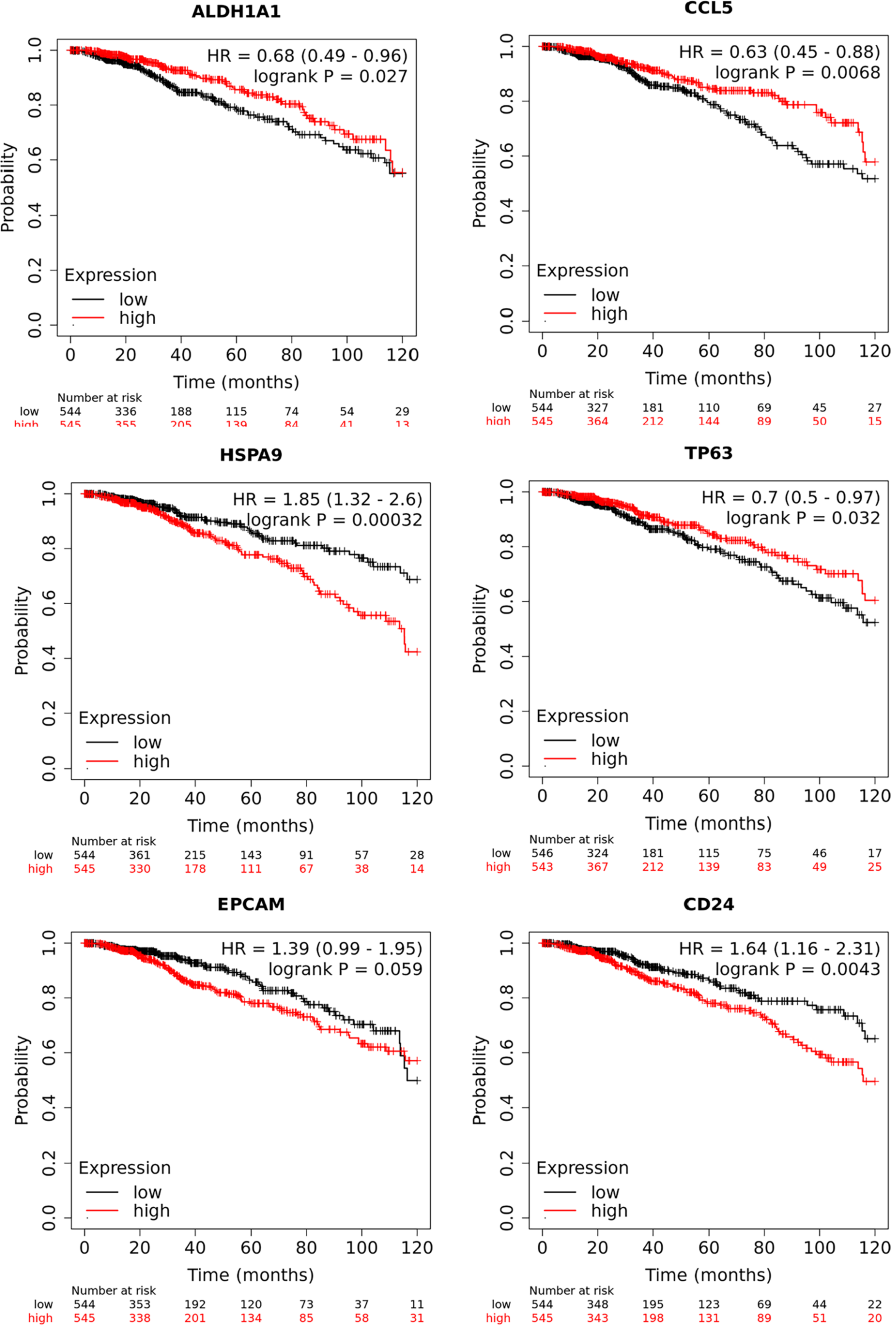
Overall survival Kaplan–Meier curves of statistically significant (*P* < 0.05) and borderline significance ALDH1 A1, CCL5, HSPA9, TP63, EPCAM, and CD24 CSC genes analyzed using KMplot (Lanczky and Gyorffy 2021). This data is based on RNA-seq data of breast cancer patients from the TCGA cohort. Patients are categorized into high and low expression based on the median expression scores, and a *P*-value of less than 0.05 is considered as statistically significant

**Fig. 5:**
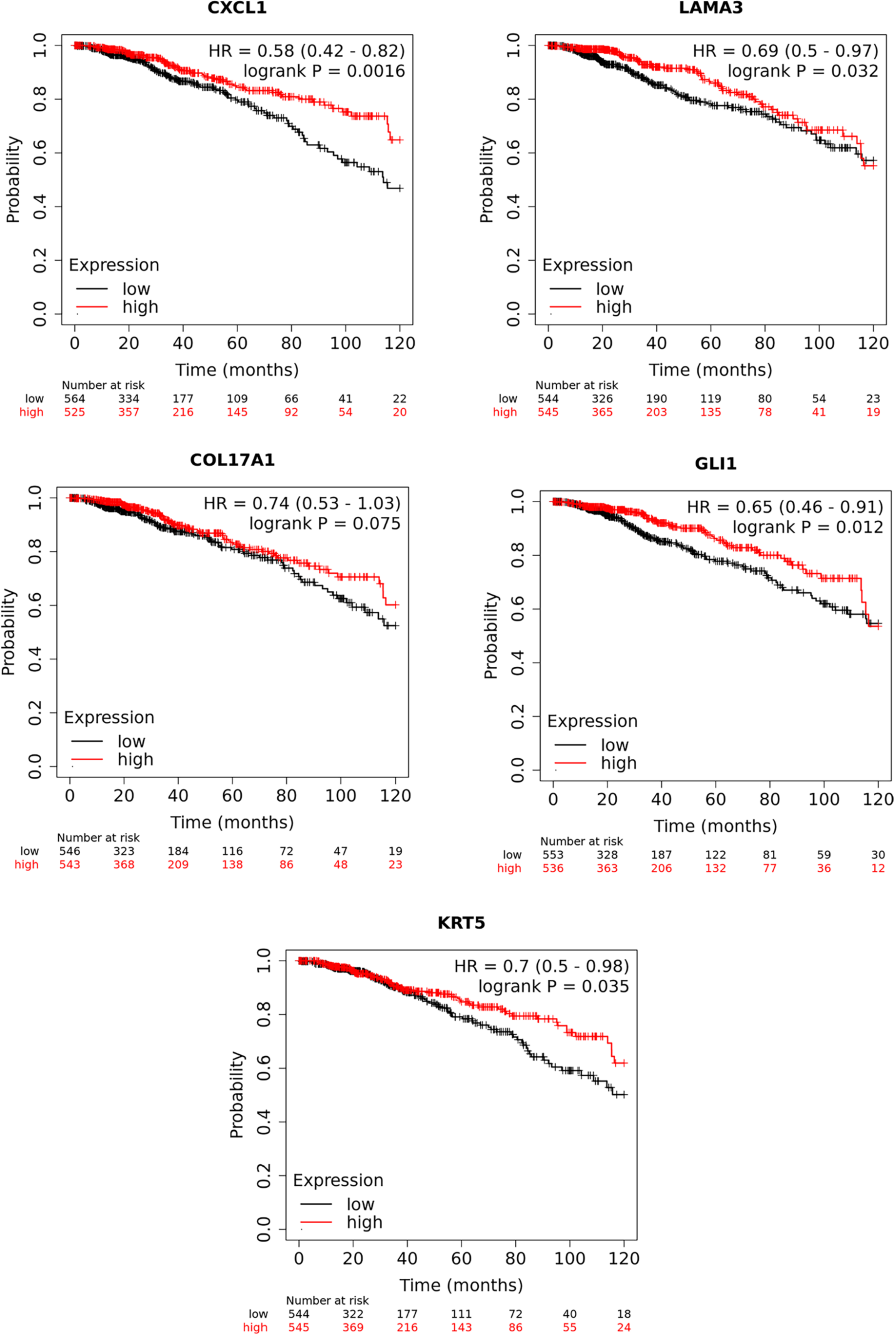
Overall survival Kaplan–Meier curves of statistically significant (*P* < 0.05) and borderline significance CXCL1, LAMA3 CoL17 A1, GLI1, and KRT5 CSC genes analyzed using KMplot (Lanczky and Gyorffy 2021). This data is based on RNA-seq data of breast cancer patients from the TCGA cohort. Patients are categorized into high and low expression based on the median expression scores, and a *P*-value of less than 0.05 is considered as statistically significant

Interactions between mTOR and CSC genes are depicted in a gene regulatory network. By utilizing this network, we may better understand the intricate relationship between these genes and draw conclusions about potential treatments and cause disease (Fig. 3). In addition, there has been evidence linking cancer metastasis and chemo-resistance to markers specific to CSCs, including CD44, CD24, ALDH1, and CD133 (Sheridan et al. 2006; Fraszczak and Barczynski 2023; Izycka et al. 2023). Moreover, circulating BCSC markers, EpCAM, CD44, CD24, ALDH1, CD133, and PIWIL2 have also been identified as critical determinants of prognosis, diagnosis, and prediction of response (Table 1) (Mansoori et al. 2017; Kong et al. 2018; Kehoe et al. 2015). Patients with BC who exhibit these CSC markers at high levels have a very low chance of overall survival (Figs. 4–5). CD44 is the most common tumor biomarker used in BC stratification. It cooperates with RTK and regulates BCSC proliferation, adhesion, and migration (Yousefnia et al. 2020; Liu et al. 2014).

**Table 1:** Major stem cell marker and function in breast cancer

Markers	Functions	Reference
CD44 and CD24	Supports tumor growth, epithelial–mesenchymal transition (EMT), and drug resistance	(Li et al. 2017; Xu et al. 2016)
Aldehyde dehydrogenase 1	Promotes angiogenesis Drives epithelial–mesenchymal transition (EMT) via TWIST1 and MUC1-C pathways Modulates the tumor microenvironment and tumor vascularization	(Althobiti et al. 2020; Ciccone et al. 2018; Wei et al. 2022; Raghunathan et al. 1988)
ABCG2	Confers multidrug resistance, enhances sphere-forming ability	(Nakanishi and Ross 2012; Sicchieri et al. 2015)
CD133	Plays a major role in tumor progression and metastasis, and confers treatment resistance	(Li et al. 2017; Brugnoli et al. 2019)
CD49f	Stemness maintenance, tumor initiation and progression Metastasis promotion	(Barbieri et al. 2017; Lee et al. 2019; Xia and Xu 2015; Mohammed et al. 2013)
LGR5	Has the ability to self-renew spheres and tumorigenicity, activate Wnt/β-catenin signaling, and increase the stemness of breast cancer cells	(Lee et al. 2021; Montazer et al. 2023; Yang et al. 2015)
CD70	Metastasis promotion, enhanced tumorigenicity, and marker for aggressive phenotype	(Kitajima et al. 2018; Liu et al. 2018)
EpCAM (CD326)	Facilitates proliferation, differentiation, and cell signaling; linked to tumor progression	(Lehr 1996)
Sox2	Regulates pluripotency and is necessary for mammosphere formation	(Johnson et al. 2019; Leis et al. 2012; Shim 2014)
Oct4	Maintains stemness and is associated with tamoxifen resistance	(Gwak et al. 2017)
Nanog	Enhances self-renewal and correlates with tumor size, grade, stage, and poor survival	(Emadian Saravi et al. 2019; Shan et al. 2012; Wang et al. 2014)
ZEB1	Drives epithelial-to-mesenchymal transition (EMT), increases CD44^+^/CD24^−^ population	(Feldker et al. 2020; Murray et al. 2016; Shivhare et al. 2023)

Several signaling pathways including PI3 K/AKT pathways are known to be stimulated by CD44 (Herishanu et al. 2011; Vadhan et al. 2022; Ahmad et al. 2023). Bai et al. showed that the downregulation of hypoxia-inducible factor 2 alpha (HIF-2α) expression inhibits the stemness of BC cells and promotes apoptosis mediated by CD44/PI3 K/AKT/mTOR signaling pathway (Bai et al. 2020). Studies have shown that inhibition of CD44 alters BCSCs’ properties including tumor initiation, adhesion, metastasis, and treatment resistance (Al-Othman et al. 2020; Yang et al. 2020). PI3 K/AKT/mTOR signaling pathway influences CSCs’ properties and EMT phenotype associated with aggressive BC in vitro and in vivo. The first cohort study indicated that tumor samples with *PIK3 CA* mutations were associated with high PI3 K/AKT/mTOR signaling and stemness scores (Madsen et al. 2021). Because of their limited expression, the small tissue samples available, and the fact that BCSCs are not always easy to identify, indicators mediated by the PI3 K/AKT/mTOR pathway continue to provide a challenge to their identification.

## PI3 K/AKT/mTOR mediated regulation of epithelial-to-mesenchymal transition markers

During EMT, epithelial cells change from an epithelial phenotype to a fibroblastic phenotype, leading the cancer cells to lose epithelial markers (E-cadherin, α-catenin, and γ-catenin) and to gain mesenchymal markers (N-cadherin fibronectin, and vimentin) (Zhang et al. 2025; Kanwal et al. 2025; Blaszczak et al. 2025). EMT regulates metastasis, invasion, chemoresistance, and immunosuppression in BCSCs (Blaszczak et al. 2025; Yuan et al. 2024). BCSCs are characterized by a more dormant/quiescent mesenchymal-like state and CD44^+^CD24^−^/low expression, which are placed at the tumor edge and form micro-metastases at distant sites. The other type of BCSCs is characterized by a proliferative/epithelial-like state and ALDH activity which is situated at the tumor center and restored epithelial-like state (Bushnell et al. 2021). BCSCs can induce EMT in human mammary epithelial cells (Mani et al. 2008; Liaghat et al. 2024). Moreover, Hennessy et al. indicated that basal B/claudin-low BC cells expressed both EMT and stem cell surface markers (Hennessy et al. 2009). Another study showed that overexpression of serine/threonine kinase (PIM1) induced EMT and BCSC properties; however, knockdown of PIM1 downregulated the expression of EMT-related transcription factors (Snail, TWIST) and mesenchymal marker N-cadherin (Gao et al. 2019), thereby clearly indicating the importance of EMT and BCSCs in synergistically promoting BC metastasis. EMT markers are associated with reduced immune recognition and increased expression of immune checkpoint proteins, while CSCs exhibit resistance to immune-mediated destruction. CSCs evade immune detection through PI3 K/AKT/mTOR pathway activation, which downregulates MHC molecules, promotes immunosuppressive cytokines (e.g., TGF-β, IL-10), and upregulates immune checkpoints like PD-L1 (Pan et al. 2025; Wu et al. 2023). Targeting this pathway may disrupt CSC-mediated immune escape, suggesting potential synergy with immunotherapy (Ibrahim et al. 2025; Hashemi et al. 2024).

The PI3 K/AKT/mTOR (PAM) pathway has been implicated to play a pivotal role in BC progression via governing the maintenance of BCSCs and their EMT properties (Chang et al. 2013; Hong et al. 2024). A previous study on HER2-positive BC reported that HER2 is involved in inducing the expression of stem cell–related genes (OCT3/4, NOTCH1, NOTCH2, JAG1), thus leading to activation of the PI3 K/AKT pathway (Korkaya et al. 2008; DiNatale et al. 2022). Therefore, the dysregulation of HER2 leads to improved AKT phosphorylation in the ALDH-positive population of BCSCs (Alanazi and Khan 2016). PI3 K/AKT pathway activates the runt-related transcription factor 2, leading to carcinogenicity, progression, and EMT in BCSCs (Fritz et al. 2020). However, understanding the exact mechanisms of PI3 K/AKT/mTOR-mediated EMT signaling in BCSCs is a challenge.

## Therapeutic approaches for targeting PI3 K/AKT/mTOR pathway in BCSCs

Deregulation of the PI3 K/AKT/mTOR molecular pathway is associated with the progression of various cancers (Hussain et al. 2015; Ahmed et al. 2014; Du FY et al. 2019); thus, agents such as inhibitors/antagonists/agonists were structurally designed and formulated to target the above cascade in multiple cancers and CSCs, including BC. Although a small percentage of small molecule inhibitors targeting PI3 K/AKT/mTOR are under investigation, a handful has been used in clinics. Here in the segment below, we have summarized the molecules that have the potential to control the growth of CSCs and may find a special place in translational research.

The pan-PI3 K inhibitor dihydro benzofuran-imidazolium salt, B591, was reported to have a strong inhibitory effect on class I PI3 K isoforms by preventing the activation of PI3 K/AKT/mTOR signaling pathways in MDA-MB-231 and SUM-159PT cell lines. In addition, this inhibitor proved to be more effective at reducing CSCs survival and eradicating CSCs than bulk tumor cell populations and significantly eradicated CSCs when combined with paclitaxel. In an in vivo mouse xenograft model of human BC, B591 dramatically decreased tumor-initiating capacity, demonstrating that it primarily lowered CSC levels (Zhou et al. 2019) 30,635,656.

Yu et al. (Yu et al. 2016) found that when NVP-BKM120, a PI3 K inhibitor, was combined with trastuzumab or RAD001 (everolimus), the potential to target BCSCs increased. The results were subsequently corroborated using xenograft mouse models, which showed that combining BKM120 with trastuzumab or RAD001 reduced the mammosphere-forming efficiency (MFE) (Yu et al. 2016; Maira et al. 2012). Consistent with the findings above, another study revealed that NVP-BKM120 (BKM120) significantly reduced PI3 K, AKT1, and S6 expression, inhibiting BCSCs-CYP19 growth, migration, and colony formation (Hu et al. 2015). In conjunction with letrozole, PI3 K, AKT1, and S6 expression levels were reduced, impacting the development and proliferation of BCSCs-CYP19 (Liu et al. 2019; Chae and Kim 2021; Hakeem et al. 2024).

AKT/mTOR and p38 MAPK signaling pathways are both modulated by the steroid saponin dioscin, which inhibits the development of BC cells (Chae and Kim 2021). It specifically targets BCSCs in MDA-MB-231 and MCF-7 models, resulting in G2/M and G0/G1 cell cycle arrest, respectively (Chae and Kim 2021). By blocking the PI3 K/Akt/mTOR axis and reducing cancer stem cell indicators such as ALDH, piperine and doxorubicin in combination helps to overcome chemoresistance in triple-negative breast cancer (TNBC) (Hakeem et al. 2024). Concurrently, wortmannin—a broad-spectrum inhibitor of PI3 K/Akt/mTOR—enhances the antiproliferative effects of mesenchymal stem cell–conditioned medium (MSC-CM) in BC cells. This combination inhibits the PI3 K/AKT/mTOR signaling pathways, inducing apoptosis and autophagy-mediated cell death (Ismail et al. 2025).

Anindita Chakrabarty and colleagues (Chakrabarty et al. 2013) investigated the effects of trastuzumab alone and in combination with the pan-PI3 K inhibitor XL147. The combination group lowered proliferation and pAKT levels, resulting in the death of trastuzumab-resistant HR5 and HR6 cell lines. The combination outperformed XL147 in anticancer activity in a trastuzumab-resistant tumor xenograft model. Trastuzumab, in combination with XL147, also decreased CSCs in trastuzumab-resistant tumors (Chakrabarty et al. 2013). Treating trastuzumab-resistant cell lines (MDA-MB-453 and JIMT-1) with the PI3 K inhibitor LY-294002 reduced PI3 K/AKT signaling in a HER2-dependent manner, resulting in a significant reduction in the number of ALDH + BCSCs (Korkaya et al. 2008). Similar results were observed with LY-294002 against trastuzumab-resistant cell lines (Korkaya et al. 2008).

Quercetin, a naturally occurring chemical, reduced clone formation and mammosphere development in CD44^+^/CD24 CSCs. Furthermore, quercetin reduced BCL2 and cyclin D1 expression levels via blocking the PI3/AKT/mTOR pathway (Li et al. 2018). A study by Yi Wang et al. (Wang et al. 2022) discovered that the combination of carboplatin (CBP) and thioridazine (THZ), an antipsychotic medication, inhibited CSC proliferation by inhibiting the PI3 K/AKT/mTOR pathway. Another study found that perifosine, an AKT inhibitor, effectively altered the stemness phenotype in tamoxifen-resistant BC cells (Farahmand et al. 2018). While there are a few targeted therapeutic compounds that show promise, these candidates are still in the early stages and require further investigation through in vitro experiments to comprehend their full potential.

Aberrant activation of PI3/AKT/mTOR causes activation or suppression of various protein molecules in numerous cancers, representing it as attractive therapeutic targets in multiple malignancies. Despite available targeted therapies and clinical experience, the risk for therapeutic resistance, side effects, and associated toxicities cannot be neglected, thereby limiting its clinical application. In addition, many cross-talks occurring between the PI3 K/AKT/mTOR signaling pathway and other pathways must be considered while designing effective therapeutic molecules. The drugs used in combination therapy for targeting multiple ways in solid malignancies need to have thorough follow-up and observation. Therefore, further in-depth studies are warranted to identify precise and safe therapeutic targeted agent/s to ascertain clinical benefits further.

Preclinical validation of an anticancer candidate takes place before the enrollment of participants in clinical trials to assess safety and efficacy in human subjects. Unfortunately, positive outcomes in preclinical studies may not necessarily indicate a positive outcome in clinical trials. It is common practice in preclinical in vitro investigations to subject the candidate of interest to continuous high-concentration exposure. Furthermore, compared to preclinical studies, clinical trials are somewhat costly. Immunotherapy has been used to treat different types of cancer, but not as often as surgery or more standard treatments like chemotherapy or radiation therapy. Although immunotherapy may yield positive outcomes, not every patient exhibits a response to treatment. Additionally, certain patients receiving a combination of immunotherapeutic agents may manifest immune-related disorders (Wang and Minden 2022). Table 2 displays a list of the therapeutic candidates and Table 3 summarizes combinatorial approaches that have been used in clinical trials.

**Table 2:** Therapeutic agents in clinical trials that target PI3 K/AKT/mTOR pathway

**Therapeutic agent**	**Target**	**Mechanism of action**	**Status/clinical trials**	**Clinical trial number**
Buparlisib (BKM120)	PI3 K (pan-PI3 K inhibitor)	Inhibits all class I PI3 K isoforms, reducing BCSC survival and self-renewal	Phase II/III trials in breast cancer	NCT01610284, NCT01633060, NCT01790932
Alpelisib (BYL719	PI3 Kα (p110α isoform)	Selective inhibition of PI3 Kα, suppressing BCSC proliferation and tumor growth	FDA approved for PIK3 CA-mutated breast cancer	NCT02437318
MK-2206	AKT	Allosteric AKT inhibitor, reduces BCSC stemness and enhances chemotherapy sensitivity	Phase II trials in breast cancer	NCT01277757
Ipatasertib (GDC-0068)	AKT	ATP-competitive AKT inhibitor, targets BCSC survival and tumor progression	Phase II/III trials in breast cancer	NCT02301988, NCT04464174
Everolimus (RAD001)	mTORC1	mTORC1 inhibitor, reduces BCSC self-renewal and tumorigenicity	FDA approved for hormone receptor–positive breast cancer	NCT00863655
Temsirolimus (CCI-779)	mTORC1	mTORC1 inhibitor, suppresses BCSC proliferation and survival	FDA approved for advanced breast cancer	NCT00062751
Dactolisib (BEZ235)	PI3 K/mTOR (dual inhibitor)	Dual inhibition of PI3 K and mTOR, targeting BCSC stemness and tumor growth	Phase I/II trials in breast cancer	NCT01288092, NCT01508104
Gedatolisib (PF-05212384)	PI3 K/mTOR (dual inhibitor	Dual inhibition of PI3 K and mTOR, targeting BCSC stemness and tumor growth	Phase I/II trials in breast cancer	NCT02626507, NCT01920061
Capivasertib (AZD5363)	PI3 K/mTOR (dual inhibitor)	Dual inhibitor of PI3 K and mTOR, reduces BCSC survival and tumor progression	Phase II trials in breast cancer	NCT01992952, NCT01625286, NCT03375880
Capivasertib (AZD5363)	AKT	Selective AKT inhibitor, targets BCSC stemness and enhances therapy response	Phase III trials in breast cancer	NCT04862663
LY294002	PI3 K	Broad PI3 K inhibitor, reduces BCSC self-renewal and tumorigenicity	Preclinical studies	Not applicable

**Table 3:** Combinatorial approaches under clinical trial for treatment of breast cancer

Clinical trials	Status	Combination therapy components
NCT02246621	Active, not recruiting—phase III	Abemaciclib + fulvestrant/anastrozole/letrozole
NCT03155997	Active, not recruiting—phase III	Abemaciclib + tamoxifen/anastrozole/letrozole
NCT01231659	Completed—phase II	Everolimus + letrozole
NCT01082068	Completed—phase II	Pilaralisib/voxtalisib + letrozole
NCT01491737	Completed	Anastrozole/letrozole + pertuzumab/trastuzumab
NCT02734004	Active, not recruiting—phase II	Olaparib + durvalumab
NCT03036488	Active, not recruiting, phase III	Pembrolizumab + nab-paclitaxel/paclitaxel/gemcitabine + carboplatin
NCT02425891	Completed—phase III	Atezolizumab + nab-paclitaxel
NCT02614794	Completed—phase II	Trastuzumab + capecitabine + tucatinib
NCT01992952	Unknown status	AZD5363 + fulvestrant
NCT01942135	Completed—phase III	Palbociclib + fulvestrant
NCT02422615	Completed—phase III	Ribociclib + fulvestrant
NCT02456857	Completed—phase II	Doxorubicin/bevacizumab/everolimus
NCT01281696	Completed—phase II	Bevacizumab + etoposide/cisplatin
NCT02000622	Active, not recruiting, phase III	Olaparib + capecitabine/vinorelbine/eribulin
NCT01698918	Completed—phase II	Everolimus + letrozole
NCT02657889	Completed—phase II	Niraparib + pembrolizumab
NCT03036488	Active, not recruiting, phase III	Pembrolizumab + chemotherapy
NCT03125902	Active, not recruiting, phase III	Atezolizumab + chemotherapy
NCT01942135	Completed, phase III	Palbociclib + fulvestrant
NCT01958021	Completed, phase III	Letrozole + ribociclib
NCT02657889	Completed, phase II	Niraparib + pembrolizumab
NCT01584648	Completed, phase III	Dabrafenib + trametinib
NCT02993523	Active, not recruiting, phase III	Venetoclax + azacitidine
NCT03840200	Completed, phase II	Ipatasertib + rucaparib
NCT0430549	Active, not recruiting, phase III	Capivasertib + fulvestrant

## Conclusion

Breast cancer stem cells (BCSCs) constitute a vital subset of tumor-initiating cells that accelerate tumor development, metastasis, resistance, and recurrence. These cells exhibit strong self-renewal and differentiation abilities, facilitated by dysregulated signaling pathways, notably the PI3 K/AKT/mTOR axis, which promotes their survival, proliferation, and EMT.

Targeting the PI3 K/AKT/mTOR pathway has become a viable approach to eradicate BCSCs; nonetheless, difficulties like intrinsic and acquired resistance remain. Integrating PI3 K/AKT/mTOR inhibitors with drugs that target alternative cancer stem cell–related pathways, epithelial–mesenchymal transition regulators, or the tumor microenvironment may enhance treatment effectiveness. Clinical trials are now being conducted on a number of PI3 K/AKT/mTOR inhibitors for breast cancer, both alone and in combination. In addition to creating next-generation inhibitors with enhanced selectivity and less toxicity, future research should concentrate on finding predictive biomarkers to stratify individuals who are most likely to benefit from these treatments. Targeting PI3 K/AKT/mTOR signaling in breast cancer stem cells presents considerable potential to enhance clinical outcomes and long-term survival in breast cancer patients.

## Data Availability

All source data for this work (or generated in this study) are available upon reasonable request.

## Abbreviations

ALDH: Aldehyde dehydrogenase
BC: Breast cancer
BCSC: Breast cancer stem cells
BRCA1: Breast cancer gene 1
BRCA2: Breast cancer gene 2
CBP: Carboplatin
CD24: Cluster of differentiation 24
CD44: Cluster of differentiation 44
CDK1: Cyclin-dependent kinase1
CSCs: Cancer stem cells
EMT: Epithelial–mesenchymal transition
EpCAM: Epithelial cellular adhesion molecule
ER: Estrogen receptor
FOXO: Forkhead box transcription factors
GPCRs: G protein–coupled receptors
GSK-3β: Glycogen synthase kinase-3beta
HER2: Human epidermal growth factor receptor 2
mSLT8: Mammalian lethal with SEC13 protein 8
mTOR C2: MTOR complex 2
NF-κB: Nuclear factor kappa-light-chain-enhancer of activated B cells
Oct4: Octamer-binding transcription factor 4
PDK1: Phosphoinositide-dependent protein kinase
PH: Pleckstrin homology interaction domain
PI3K: Phosphatidylinositol 3-kinase
PIP2: Phosphatidylinositol(4,5)-bisphosphate
PIP3: Phosphatidylinositol(3,4,5)-trisphosphate
PIK3CA: Phosphatidylinositol-4,5-bisphosphate 3-kinase catalytic subunit alpha
PR: Progesterone receptor
PTEN: Phosphatase and tensin homolog
Raptor: Regulator associated protein of mTOR
RTK: Receptor tyrosine kinase
Sin1: Stress-activated protein kinase interacting protein 1
Sox2: SRY-box transcription factor 2
TNBC: Triple-negative breast cancer
TSC: Tuberous sclerosis complex
TKD: Tyrosine kinase domain
4EBP1: Eukaryotic translation initiation factor 4E (eIF4E)-binding protein 1
DEPTOR: DEP domain-containing mTOR-interacting protein
RICTOR: Rapamycinin sensitive companion of mammalian target of rapamycin
PDK-1: 3-Phosphoinositide-dependent kinase 1

## Declarations

### Name of principal scientist

Shahab Uddin.

## References

[CR1] Abdelaziz N et al (2023) Epigenetic inhibitors and their role in cancer therapy. Int Rev Cell Mol Biol 380:211–251 37657859 10.1016/bs.ircmb.2023.04.005

[CR2] de Oliveira WAA et al (2021) Wnt/beta-catenin inhibition disrupts carboplatin resistance in isogenic models of triple-negative breast cancer. Front Oncol 11:705384 10.3389/fonc.2021.705384 PMC8340846 34367990

[CR3] Adon T et al (2025) Structural requirements of isoform-specific inhibitors of Akt: implications in the development of effective cancer treatment strategies. Eur J Med Chem 287:117334 39904143 10.1016/j.ejmech.2025.117334

[CR4] Agrawal M, Agrawal SK, Chopra K (2025) Overcoming drug resistance in ovarian cancer through PI3K/AKT signaling inhibitors. Gene 948:149352 39988188 10.1016/j.gene.2025.149352

[CR5] Ahmad SMS et al (2023) ITGB1BP1, a novel transcriptional target of CD44-downstream signaling promoting cancer cell invasion. Breast Cancer (Dove Med Press) 15:373–380 37252376 10.2147/BCTT.S404565 PMC10225144

[CR6] Ahmed M et al (2014) High prevalence of mTOR complex activity can be targeted using Torin2 in papillary thyroid carcinoma. Carcinogenesis 35(7):1564–1572 24583924 10.1093/carcin/bgu051

[CR7] Alanazi IO, Khan Z (2016) Understanding EGFR signaling in breast cancer and breast cancer stem cells: overexpression and therapeutic implications. Asian Pac J Cancer Prev 17(2):445–453 26925626 10.7314/apjcp.2016.17.2.445

[CR8] Albrektsen G et al (2005) Breast cancer risk by age at birth, time since birth and time intervals between births: exploring interaction effects. Br J Cancer 92(1):167–175 15597097 10.1038/sj.bjc.6602302 PMC2361726

[CR9] Al-Dhfyan A, Alhoshani A, Korashy HM (2017) Aryl hydrocarbon receptor/cytochrome P450 1A1 pathway mediates breast cancer stem cells expansion through PTEN inhibition and beta-catenin and Akt activation. Mol Cancer 16(1):14 28103884 10.1186/s12943-016-0570-y PMC5244521

[CR10] Al-Hajj M et al (2003) Prospective identification of tumorigenic breast cancer cells. Proc Natl Acad Sci U S A 100(7):3983–3988 12629218 10.1073/pnas.0530291100 PMC153034

[CR11] Al-Othman N et al (2020) Role of CD44 in breast cancer. Breast Dis 39(1):1–13 31839599 10.3233/BD-190409

[CR12] Althobiti M et al (2020) The prognostic significance of ALDH1A1 expression in early invasive breast cancer. Histopathology 77(3):437–448 32369651 10.1111/his.14129

[CR13] Asati V, Mahapatra DK, Bharti SK (2016) PI3K/Akt/mTOR and Ras/Raf/MEK/ERK signaling pathways inhibitors as anticancer agents: structural and pharmacological perspectives. Eur J Med Chem 109:314–341 26807863 10.1016/j.ejmech.2016.01.012

[CR14] Bai J et al (2020) HIF-2α regulates CD44 to promote cancer stem cell activation in triple-negative breast cancer via PI3K/AKT/mTOR signaling. World Journal of Stem Cells 12(1):87 32110277 10.4252/wjsc.v12.i1.87 PMC7031759

[CR15] Barbieri NL et al (2017) FNR regulates the expression of important virulence factors contributing to the pathogenicity of avian pathogenic *Escherichia coli*. Front Cell Infect Microbiol 7:265 28690981 10.3389/fcimb.2017.00265 PMC5481319

[CR16] Berrino C, Omar A (2024) Unravelling the mysteries of the Sonic Hedgehog pathway in cancer stem cells: activity, crosstalk and regulation. Curr Issues Mol Biol 46(6):5397–5419 38920995 10.3390/cimb46060323 PMC11202538

[CR17] Beziaud L et al (2023) IFNgamma-induced stem-like state of cancer cells as a driver of metastatic progression following immunotherapy. Cell Stem Cell 30(6):818–831 37267916 10.1016/j.stem.2023.05.007

[CR18] Blaszczak E et al (2025) Triple-negative breast cancer progression and drug resistance in the context of epithelial-mesenchymal transition. Cancers (Basel) 17(2) 10.3390/cancers17020228 PMC11764116 39858010

[CR19] Breuer K et al (2013) InnateDB: systems biology of innate immunity and beyond--recent updates and continuing curation. Nucleic Acids Res 41(Database issue):D1228–33 10.1093/nar/gks1147 PMC3531080 23180781

[CR20] Brewer HR et al (2017) Family history and risk of breast cancer: an analysis accounting for family structure. Breast Cancer Res Treat 165(1):193–200 28578505 10.1007/s10549-017-4325-2 PMC5511313

[CR21] Brugnoli F et al (2019) CD133 in breast cancer cells: more than a stem cell marker. J Oncol 2019:7512632 31636668 10.1155/2019/7512632 PMC6766124

[CR22] Bushnell GG et al (2021) Breast cancer dormancy: need for clinically relevant models to address current gaps in knowledge. NPJ Breast Cancer 7(1):1–12 34050189 10.1038/s41523-021-00269-x PMC8163741

[CR23] Chae WO, Kim GD (2021) *Dioscin* decreases breast cancer stem-like cell proliferation via cell cycle arrest by modulating *p38* mitogen-activated protein kinase and AKT/mTOR signaling pathways. J Cancer Prev 26(3):183–194 34703821 10.15430/JCP.2021.26.3.183 PMC8511578

[CR24] Chakrabarty A et al (2013) Trastuzumab-resistant cells rely on a HER2-PI3K-FoxO-survivin axis and are sensitive to PI3K inhibitors. Cancer Res 73(3):1190–1200 23204226 10.1158/0008-5472.CAN-12-2440 PMC3563941

[CR25] Chang W-W et al (2013) The expression and significance of insulin-like growth factor-1 receptor and its pathway on breast cancer stem/progenitors. Breast Cancer Res 15(3):1–16 10.1186/bcr3423 PMC3706809 23663564

[CR26] Chen Y et al (2011) Sonic Hedgehog dependent phosphorylation by CK1alpha and GRK2 is required for ciliary accumulation and activation of smoothened. PLoS Biol 9(6):e1001083 21695114 10.1371/journal.pbio.1001083 PMC3114773

[CR27] Chen X et al (2019) Physical activity and risk of breast cancer: a meta-analysis of 38 cohort studies in 45 study reports. Value Health 22(1):104–128 30661625 10.1016/j.jval.2018.06.020

[CR28] Chu X et al (2024) Cancer stem cells: advances in knowledge and implications for cancer therapy. Signal Transduct Target Ther 9(1):170 38965243 10.1038/s41392-024-01851-y PMC11224386

[CR29] Ciccone V et al (2018) Stemness marker ALDH1A1 promotes tumor angiogenesis via retinoic acid/HIF-1alpha/VEGF signalling in MCF-7 breast cancer cells. J Exp Clin Cancer Res 37(1):311 30541574 10.1186/s13046-018-0975-0 PMC6291966

[CR30] Damiani D, Tiribelli M (2024) ATP-binding cassette subfamily G member 2 in acute myeloid leukemia: a new molecular target? Biomedicines 12(1) 10.3390/biomedicines12010111 PMC10813371 38255216

[CR31] DiNatale A et al (2022) Regulation of tumor and metastasis initiation by chemokine receptors. J Cancer 13(11):3160–3176 36118530 10.7150/jca.72331 PMC9475358

[CR32] Diop A et al SH2 domains: folding, binding and therapeutical approaches. Int J Mol Sci 23(24) 10.3390/ijms232415944 PMC9783222 36555586

[CR33] Du FY et al (2019) Targeting cancer stem cells in drug discovery: current state and future perspectives. World J Stem Cells 11(7):398–420 31396368 10.4252/wjsc.v11.i7.398 PMC6682504

[CR34] Dworakowska D et al (2009) Activation of RAF/MEK/ERK and PI3K/AKT/mTOR pathways in pituitary adenomas and their effects on downstream effectors. Endocr Relat Cancer 16(4):1329–1338 19620247 10.1677/ERC-09-0101

[CR35] Emadian Saravi O et al (2019) Immunohistochemical expression of Nanog and its relation with clinicopathologic characteristics in breast ductal carcinoma. Iran Biomed J 23(3):184–189 30220190 10.29252/.23.3.184 PMC6462300

[CR36] Erol A et al (2019) Sex hormones in alcohol consumption: a systematic review of evidence. Addict Biol 24(2):157–169 29280252 10.1111/adb.12589 PMC6585852

[CR37] Eve L et al (2020) Exposure to endocrine disrupting chemicals and risk of breast cancer. Int J Mol Sci 21(23) 10.3390/ijms21239139 PMC7731339 33266302

[CR38] Fanucci K et al (2024) Practical treatment strategies and novel therapies in the phosphoinositide 3-kinase (PI3K)/protein kinase B (AKT)/mammalian target of rapamycin (mTOR) pathway in hormone receptor-positive/human epidermal growth factor receptor 2 (HER2)-negative (HR+/HER2-) advanced breast cancer. ESMO Open 9(12):103997 39674130 10.1016/j.esmoop.2024.103997 PMC11699375

[CR39] Farahmand L et al (2018) *Stemness* phenotype in tamoxifen resistant breast cancer cells may be induced by interactions between receptor tyrosine kinases *and ERalpha-66*. Recent Pat Anticancer Drug Discov 13(3):302–307 29512469 10.2174/1574892813666180305164634

[CR40] Feldker N et al (2020) Genome-wide cooperation of EMT transcription factor ZEB1 with YAP and AP-1 in breast cancer. EMBO J 39(17):e103209 32692442 10.15252/embj.2019103209 PMC7459422

[CR41] Fraszczak K Barczynski B (2023) The role of cancer stem cell markers in ovarian cancer. Cancers (Basel) 16(1) 10.3390/cancers16010040 PMC10778113 38201468

[CR42] Fritz AJ et al (2020) RUNX transcription factor mediated control of breast cancer stem cells. J Cell Physiol 235(10):7261 32180230 10.1002/jcp.29625 PMC7415511

[CR43] Gao X et al (2019) PIM1 is responsible for IL-6-induced breast cancer cell EMT and stemness via c-myc activation. Breast Cancer 26(5):663–671 30989585 10.1007/s12282-019-00966-3 PMC6694096

[CR44] Garcia-Viloca M et al (2022) Molecular insights into the regulation of 3-phosphoinositide-dependent protein kinase 1: modeling the interaction between the kinase and the pleckstrin homology domains. ACS Omega 7(29):25186–25199 35910176 10.1021/acsomega.2c02020 PMC9330272

[CR45] Garg P et al (2025) Strategic advancements in targeting the PI3K/AKT/mTOR pathway for breast cancer therapy. Biochem Pharmacol 236:116850 40049296 10.1016/j.bcp.2025.116850

[CR46] Glaviano A et al (2023) PI3K/AKT/mTOR signaling transduction pathway and targeted therapies in cancer. Mol Cancer 22(1):138 37596643 10.1186/s12943-023-01827-6 PMC10436543

[CR47] Gozzelino L et al (2020) PI(3,4)P2 Signaling in cancer and metabolism. Front Oncol 10:360 32296634 10.3389/fonc.2020.00360 PMC7136497

[CR48] Guo Q et al (2024) NF-kappaB in biology and targeted therapy: new insights and translational implications. Signal Transduct Target Ther 9(1):53 38433280 10.1038/s41392-024-01757-9 PMC10910037

[CR49] Gwak JM et al (2017) Expression of embryonal stem cell transcription factors in breast cancer: Oct4 as an indicator for poor clinical outcome and tamoxifen resistance. Oncotarget 8(22):36305–36318 28422735 10.18632/oncotarget.16750 PMC5482656

[CR50] Hakeem AN et al (2024) Piperine enhances doxorubicin sensitivity in triple-negative breast cancer by targeting the PI3K/Akt/mTOR pathway and cancer stem cells. Sci Rep 14(1):18181 39107323 10.1038/s41598-024-65508-0 PMC11303729

[CR51] Han Z et al (2024) The function of serine/threonine-specific protein kinases in B cells. Front Immunol 15:1459527 39445011 10.3389/fimmu.2024.1459527 PMC11496051

[CR52] Hao C et al (2025) PI3K/AKT/mTOR inhibitors for hormone receptor-positive advanced breast cancer. Cancer Treat Rev 132:102861 39662202 10.1016/j.ctrv.2024.102861

[CR53] Harbeck N, Gnant M (2017) Breast cancer. Lancet 389(10074):1134–1150 27865536 10.1016/S0140-6736(16)31891-8

[CR54] Hashemi M et al (2024) Targeting autophagy can synergize the efficacy of immune checkpoint inhibitors against therapeutic resistance: new promising strategy to reinvigorate cancer therapy. Heliyon 10(18):e37376 39309904 10.1016/j.heliyon.2024.e37376 PMC11415696

[CR55] He M et al (2019) Mesenchymal stem cells-derived IL-6 activates AMPK/mTOR signaling to inhibit the proliferation of reactive astrocytes induced by hypoxic-ischemic brain damage. Exp Neurol 311:15–32 30213506 10.1016/j.expneurol.2018.09.006

[CR56] Hennessy BT et al (2009) Characterization of a naturally occurring breast cancer subset enriched in epithelial-to-mesenchymal transition and stem cell characteristics. Can Res 69(10):4116–4124 10.1158/0008-5472.CAN-08-3441 PMC2737191 19435916

[CR57] Herishanu Y et al (2011) CD44 signaling via PI3K/AKT and MAPK/ERK pathways protects CLL cells from spontaneous and drug induced apoptosis through MCL-1. Leuk Lymphoma 52(9):1758 21649540 10.3109/10428194.2011.569962 PMC3403533

[CR58] Hill DA et al (2019) Temporal trends in breast cancer survival by race and ethnicity: a population-based cohort study. PLoS ONE 14(10):e0224064 10.1371/journal.pone.0224064 PMC6812853 31647839

[CR59] Hong T et al (2024) PARP9 knockdown confers protection against chemoresistance and immune escape of breast cancer cells by blocking the PI3K/AKT pathway. Arch Med Sci 20(4):1228–1248 39439687 10.5114/aoms/161444 PMC11493048

[CR60] Hu Y et al (2015) Effects of PI3K inhibitor NVP-BKM120 on overcoming drug resistance and eliminating cancer stem cells in human breast cancer cells. Cell Death Dis 6(12):e2020 26673665 10.1038/cddis.2015.363 PMC4720896

[CR61] Hua H et al (2019) Targeting mTOR for cancer therapy. J Hematol Oncol 12(1):71 31277692 10.1186/s13045-019-0754-1 PMC6612215

[CR62] Hussain AR et al (2015) Dual targeting of mTOR activity with Torin2 potentiates anticancer effects of cisplatin in epithelial ovarian cancer. Mol Med 21(1):466–478 26023849 10.2119/molmed.2014.00238 PMC4607622

[CR63] Ibrahim A et al (2025) MDSC checkpoint blockade therapy: a new breakthrough point overcoming immunosuppression in cancer immunotherapy. Cancer Gene Ther 32(4):371–392 40140724 10.1038/s41417-025-00886-9 PMC11976280

[CR64] Ippen FM et al (2019) Targeting the PI3K/Akt/mTOR pathway with the pan-Akt inhibitor GDC-0068 in PIK3CA-mutant breast cancer brain metastases. Neuro Oncol 21(11):1401–1411 31173106 10.1093/neuonc/noz105 PMC6827829

[CR65] Ismail DF et al (2025) Impregnation of mesenchymal stem cell conditioned media with wortmannin enhanced its antiproliferative effect in breast cancer cells via PI3K/Akt/mTOR pathway. BMC Res Notes 18(1):93 40038752 10.1186/s13104-025-07124-3 PMC11877855

[CR66] Izycka N et al (2023) The prognostic value of cancer stem cell markers (CSCs) expression-ALDH1A1, CD133, CD44-for survival and long-term follow-up of ovarian cancer patients. Int J Mol Sci 24(3) 10.3390/ijms24032400 PMC9916537 36768723

[CR67] JDePolo J(2024) Molecular subtypes of breast cancer. Breast Cancer Org

[CR68] Jan A et al (2025) An update on cancer stem cell survival pathways involved in chemoresistance in triple-negative breast cancer. Future Oncol 21(6):715–735 39936282 10.1080/14796694.2025.2461443 PMC11881842

[CR69] Jhanwar-Uniyal M et al (2019) Diverse signaling mechanisms of mTOR complexes: mTORC1 and mTORC2 in forming a formidable relationship. Adv Biol Regul 72:51–62 31010692 10.1016/j.jbior.2019.03.003

[CR70] Johns LE et al (2018) Domestic light at night and breast cancer risk: a prospective analysis of 105 000 UK women in the Generations Study. Br J Cancer 118(4):600–606 29360812 10.1038/bjc.2017.359 PMC5830585

[CR71] Johnson CE et al (2019) Correction: Loss of tuberous sclerosis complex 2 sensitizes tumors to nelfinavir-bortezomib therapy to intensify endoplasmic reticulum stress-induced cell death. Oncogene 38(16):3102 30622341 10.1038/s41388-018-0598-0 PMC8075855

[CR72] Jones ME et al (2017) Smoking and risk of breast cancer in the Generations Study cohort. Breast Cancer Res 19(1):118 29162146 10.1186/s13058-017-0908-4 PMC5698948

[CR73] Kanwal R et al (2025) Exploring the role of epithelial-mesenchymal transcriptional factors involved in hematological malignancy and solid tumors: a systematic review. Cancers (Basel) 17(3) 10.3390/cancers17030529 PMC11817253 39941895

[CR74] Karami Fath M et al (2022) PI3K/Akt/mTOR signaling pathway in cancer stem cells. Pathol Res Pract 237:154010 35843034 10.1016/j.prp.2022.154010

[CR75] Katsura C et al (2022) Breast cancer: presentation, investigation and management. Br J Hosp Med (Lond) 83(2):1–7 35243878 10.12968/hmed.2021.0459

[CR76] Kearney AL et al (2021) Akt phosphorylates insulin receptor substrate to limit PI3K-mediated PIP3 synthesis. Elife 10:e66942. 10.7554/eLife.66942 PMC8277355 34253290

[CR77] Kehoe S et al (2015) Primary chemotherapy versus primary surgery for newly diagnosed advanced ovarian cancer (CHORUS): an open-label, randomised, controlled, non-inferiority trial. Lancet 386(9990):249–257 26002111 10.1016/S0140-6736(14)62223-6

[CR78] Khayatan D et al (2025) Targeting mTOR with curcumin: therapeutic implications for complex diseases. Inflammopharmacology 33:1583–1616 39955697 10.1007/s10787-025-01643-y

[CR79] Kilmister EJ, Tan ST (2025) Cancer stem cells and the renin-angiotensin system in the tumor microenvironment of melanoma: implications on current therapies. Int J Mol Sci 26(3) 10.3390/ijms26031389 PMC11818896 39941158

[CR80] Kim CW, Lee JM, Park SW (2023) Divergent roles of the regulatory subunits of class IA PI3K. Front Endocrinol (Lausanne) 14:1152579 38317714 10.3389/fendo.2023.1152579 PMC10839044

[CR81] Kinsella MD et al (2013) Immunohistochemical detection of estrogen receptor, progesterone receptor and human epidermal growth factor receptor 2 in formalin-fixed breast carcinoma cell block preparations: correlation of results to corresponding tissue block (needle core and excision) samples. Diagn Cytopathol 41(3):192–198 22611048 10.1002/dc.21815

[CR82] Kitajima S et al (2018) Hypoxia-inducible factor-2 alpha up-regulates CD70 under hypoxia and enhances anchorage-independent growth and aggressiveness in cancer cells. Oncotarget 9(27):19123–19135 29721188 10.18632/oncotarget.24919 PMC5922382

[CR83] Kong Y et al (2018) Breast cancer stem cell markers CD44 and ALDH1A1 in serum: distribution and prognostic value in patients with primary breast cancer. J Cancer 9(20):3728 30405844 10.7150/jca.28032 PMC6215997

[CR84] Korkaya H et al (2008) HER2 regulates the mammary stem/progenitor cell population driving tumorigenesis and invasion. Oncogene 27(47):6120–6130 18591932 10.1038/onc.2008.207 PMC2602947

[CR85] Korkaya H et al (2009) Regulation of mammary stem/progenitor cells by PTEN/Akt/beta-catenin signaling. PLoS Biol 7(6):e1000121 19492080 10.1371/journal.pbio.1000121 PMC2683567

[CR86] Kumar BH, Kabekkodu SP, Pai KSR (2025) Structural insights of AKT and its activation mechanism for drug development. Mol Divers. 10.1007/s11030-025-11132-7 40009150 10.1007/s11030-025-11132-7 PMC12638386

[CR87] Lanczky A, Gyorffy B (2021) Web*-*based survival analysis tool tailored for medical research (KMplot)*:* development and implementation. J Med Internet Res 23(7):e27633 34309564 10.2196/27633 PMC8367126

[CR88] Lee K, Kim S, Yang YL (2019) Preliminary study of outcome-based clinical practicum for undergraduate nursing students. Jpn J Nurs Sci 16(2):145–154 30022598 10.1111/jjns.12222

[CR89] Lee HJ et al (2021) Expression of LGR5 in mammary myoepithelial cells and in triple-negative breast cancers. Sci Rep 11(1):17750 34493772 10.1038/s41598-021-97351-y PMC8423726

[CR90] Lehr GJ (1996) Determination of diphenoxylate hydrochloride and atropine sulfate in combination drug formulations by liquid chromatography. J AOAC Int 79(6):1288–1293 8946706

[CR91] Leis O et al (2012) Sox2 expression in breast tumours and activation in breast cancer stem cells. Oncogene 31(11):1354–1365 21822303 10.1038/onc.2011.338

[CR92] Levina A et al (2022) Activation of the essential kinase PDK1 by phosphoinositide-driven trans-autophosphorylation. Nat Commun 13(1):1874 35387990 10.1038/s41467-022-29368-4 PMC8986801

[CR93] Li W et al (2017) Unraveling the roles of CD44/CD24 and ALDH1 as cancer stem cell markers in tumorigenesis and metastasis. Sci Rep 7(1):13856 29062075 10.1038/s41598-017-14364-2 PMC5653849

[CR94] Li X et al (2018) Quercetin suppresses breast cancer stem cells (CD44(+)/CD24(-)) by inhibiting the PI3K/Akt/mTOR-signaling pathway. Life Sci 196:56–62 29355544 10.1016/j.lfs.2018.01.014

[CR95] Li X et al (2020) Andrographolide enhanced radiosensitivity by downregulating glycolysis via the inhibition of the PI3K-Akt-mTOR signaling pathway in HCT116 colorectal cancer cells. J Int Med Res 48(8):300060520946169 32787737 10.1177/0300060520946169 PMC7427152

[CR96] Li Q et al (2022) Targeting the PI3K/AKT/mTOR and RAF/MEK/ERK pathways for cancer therapy. Mol Biomed 3(1):47 36539659 10.1186/s43556-022-00110-2 PMC9768098

[CR97] Li H et al (2024) Targeting PI3K family with small-molecule inhibitors in cancer therapy: current clinical status and future directions. Mol Cancer 23(1):164 39127670 10.1186/s12943-024-02072-1 PMC11316348

[CR98] Liaghat M et al (2024) The impact of epithelial-mesenchymal transition (EMT) induced by metabolic processes and intracellular signaling pathways on chemo-resistance, metastasis, and recurrence in solid tumors. Cell Commun Signal 22(1):575 39623377 10.1186/s12964-024-01957-4 PMC11610171

[CR99] Liu S et al (2014) Breast cancer stem cells transition between epithelial and mesenchymal states reflective of their normal counterparts. Stem Cell Reports 2(1):78–91 24511467 10.1016/j.stemcr.2013.11.009 PMC3916760

[CR100] Liu L et al (2018) Breast cancer stem cells characterized by CD70 expression preferentially metastasize to the lungs. Breast Cancer 25(6):706–716 29948958 10.1007/s12282-018-0880-6

[CR101] Liu Y et al (2019) NVP-BKM120 in combination with letrozole inhibit human breast cancer stem cells via PI3K/mTOR pathway. Zhonghua Yi Xue Za Zhi 99(14):1075–1080 30982255 10.3760/cma.j.issn.0376-2491.2019.14.008

[CR102] Liu L, Graff SL, Wang Y (2024) New emerging therapies targeting PI3K/AKT/mTOR/PTEN pathway in hormonal receptor-positive and HER2-negative breast cancer-current state and molecular pathology perspective. Cancers (Basel) 17(1) 10.3390/cancers17010016 PMC11718791 39796647

[CR103] Loh JJ, Ma S (2024) Hallmarks of cancer stemness. Cell Stem Cell 31(5):617–639 38701757 10.1016/j.stem.2024.04.004

[CR104] Luongo F et al (2019) PTEN tumor-suppressor: the dam of stemness in cancer. Cancers (Basel) 11(8) 10.3390/cancers11081076 PMC6721423 31366089

[CR105] Lv J et al (2015) PCDH20 functions as a tumour-suppressor gene through antagonizing the Wnt/beta-catenin signalling pathway in hepatocellular carcinoma. J Viral Hepat 22(2):201–211 24910204 10.1111/jvh.12265 PMC4344823

[CR106] Madsen RR et al (2021) Positive correlation between transcriptomic stemness and PI3K/AKT/mTOR signaling scores in breast cancer, and a counterintuitive relationship with PIK3CA genotype. PLoS Genet 17(11):e1009876 34762647 10.1371/journal.pgen.1009876 PMC8584750

[CR107] Maehama T, Dixon JE (1998) The tumor suppressor, PTEN/MMAC1, dephosphorylates the lipid second messenger, phosphatidylinositol 3,4,5-trisphosphate. J Biol Chem 273(22):13375–13378 9593664 10.1074/jbc.273.22.13375

[CR108] Maira SM et al (2012) Identification and characterization of NVP-BKM120, an orally available pan-class I PI3-kinase inhibitor. Mol Cancer Ther 11(2):317–328 22188813 10.1158/1535-7163.MCT-11-0474

[CR109] Mani SA et al (2008) The epithelial-mesenchymal transition generates cells with properties of stem cells. Cell 133(4):704–715 18485877 10.1016/j.cell.2008.03.027 PMC2728032

[CR110] Mansoori M et al (2017) Circulating cancer stem cell markers in breast carcinomas: a systematic review protocol. Syst Rev 6(1):1–6 29258583 10.1186/s13643-017-0660-y PMC5738150

[CR111] Martin GR (1981) Isolation of a pluripotent cell line from early mouse embryos cultured in medium conditioned by teratocarcinoma stem cells. Proc Natl Acad Sci U S A 78(12):7634–7638 6950406 10.1073/pnas.78.12.7634 PMC349323

[CR112] McGuire A et al (2015) Effects of age on the detection and management of breast cancer. Cancers (Basel) 7(2):908–929 26010605 10.3390/cancers7020815 PMC4491690

[CR113] Mohammed A et al (2013) Antidiabetic drug metformin prevents progression of pancreatic cancer by targeting in part cancer stem cells and mTOR signaling. Transl Oncol 6(6):649–659 24466367 10.1593/tlo.13556 PMC3890699

[CR114] Montazer F, Boozari B, Alizadeh-Navaei R (2023) Evaluation of LGR5 cancer stem cell marker expression in breast cancer and its relationship with hormonal profile and clinical pathological features. Asian Pac J Cancer Prev 24(2):467–470 36853294 10.31557/APJCP.2023.24.2.467 PMC10162602

[CR115] Mortazavi M et al (2022) Prospects of targeting PI3K/AKT/mTOR pathway in pancreatic cancer. Crit Rev Oncol Hematol 176:103749 35728737 10.1016/j.critrevonc.2022.103749

[CR116] Mostofsky E et al (2025) Effect of daily alcohol intake on sex hormone levels among postmenopausal breast cancer survivors on aromatase inhibitor therapy: a randomized controlled crossover pilot study. Breast Cancer Res 27(1):5 39789640 10.1186/s13058-024-01940-4 PMC11720806

[CR117] Mousavikia SN et al (2025) PI3K/AKT/mTOR targeting in colorectal cancer radiotherapy: a systematic review. J Gastrointest Cancer 56(1):52 39849185 10.1007/s12029-024-01160-1

[CR118] Murray IC et al (2016) Feasibility*,* accuracy, and repeatability of suprathreshold saccadic vector optokinetic perimetry. Transl vis Sci Technol 5(4):15 27617181 10.1167/tvst.5.4.15 PMC5015923

[CR119] Nakanishi T, Ross DD (2012) Breast cancer resistance protein (BCRP/ABCG2): its role in multidrug resistance and regulation of its gene expression. Chin J Cancer 31(2):73–99 22098950 10.5732/cjc.011.10320 PMC3777471

[CR120] Narod SA (2011) Hormone replacement therapy and the risk of breast cancer. Nat Rev Clin Oncol 8(11):669–676 21808267 10.1038/nrclinonc.2011.110

[CR121] Nicolini A, Ferrari P, Duffy MJ (2018) Prognostic and predictive biomarkers in breast cancer: past, present and future. Semin Cancer Biol 52(Pt 1):56–73 28882552 10.1016/j.semcancer.2017.08.010

[CR122] Orrantia-Borunda E et al (2022) Subtypes of breast cancer. In: HN Mayrovitz (ed) Breast cancer, Brisbane (AU)

[CR123] Ozsvari B et al (2017) Targeting flavin-containing enzymes eliminates cancer stem cells (CSCs), by inhibiting mitochondrial respiration: vitamin B2 (riboflavin) in cancer therapy. Aging (Albany NY) 9(12):2610–2628 29253841 10.18632/aging.101351 PMC5764395

[CR124] Pan Y et al (2025) Cancer stem cells and niches: challenges in immunotherapy resistance. Mol Cancer 24(1):52 39994696 10.1186/s12943-025-02265-2 PMC11852583

[CR125] Parise CA et al (2009) Breast cancer subtypes as defined by the estrogen receptor (ER), progesterone receptor (PR), and the human epidermal growth factor receptor 2 (HER2) among women with invasive breast cancer in California, 1999–2004. Breast J 15(6):593–602 19764994 10.1111/j.1524-4741.2009.00822.x

[CR126] Park S et al (2012) Characteristics and outcomes according to molecular subtypes of breast cancer as classified by a panel of four biomarkers using immunohistochemistry. Breast 21(1):50–57 21865043 10.1016/j.breast.2011.07.008

[CR127] Qi Y et al (2020) PTEN suppresses epithelial-mesenchymal transition and cancer stem cell activity by downregulating Abi1. Sci Rep 10(1):12685 32728066 10.1038/s41598-020-69698-1 PMC7391766

[CR128] Qiang M et al (2025) Targeting the PI3K/AKT/mTOR pathway in lung cancer: mechanisms and therapeutic targeting. Front Pharmacol 16:1516583 40041495 10.3389/fphar.2025.1516583 PMC11877449

[CR129] Raghunathan L et al (1988) Regional localization of the human genes for aldehyde dehydrogenase-1 and aldehyde dehydrogenase-2. Genomics 2(3):267–269 3397064 10.1016/0888-7543(88)90012-2

[CR130] Ragupathi A, Kim C, Jacinto E (2024) The mTORC2 signaling network: targets and cross-talks. Biochem J 481(2):45–91 38270460 10.1042/BCJ20220325 PMC10903481

[CR131] Robey RW et al (2001) Overexpression of the ATP-binding cassette half-transporter, ABCG2 (Mxr/BCrp/ABCP1), in flavopiridol-resistant human breast cancer cells. Clin Cancer Res 7(1):145–152 11205902

[CR132] Sacerdoti M et al (2023) Modulation of the substrate specificity of the kinase PDK1 by distinct conformations of the full-length protein. Sci Signal 16(789):eadd3184 10.1126/scisignal.add3184 PMC7614687 37311034

[CR133] Shan J et al (2012) Nanog regulates self-renewal of cancer stem cells through the insulin-like growth factor pathway in human hepatocellular carcinoma. Hepatology 56(3):1004–1014 22473773 10.1002/hep.25745

[CR134] Sheridan C et al (2006) CD44+/CD24-breast cancer cells exhibit enhanced invasive properties: an early step necessary for metastasis. Breast Cancer Res 8(5):1–13 10.1186/bcr1610 PMC1779499 17062128

[CR135] Shim JM (2014) The effects of wet heat and dry heat on the gait and feet of healthy adults. J Phys Ther Sci 26(2):183–185 24648627 10.1589/jpts.26.183 PMC3944284

[CR136] Shivhare S et al (2023) ZEB1 potentiates chemoresistance in breast cancer stem cells by evading apoptosis. Biochim Biophys Acta Mol Cell Res 1870(7):119528 37356459 10.1016/j.bbamcr.2023.119528

[CR137] Sicchieri RD et al (2015) ABCG2 is a potential marker of tumor-initiating cells in breast cancer. Tumour Biol 36(12):9233–9243 26091795 10.1007/s13277-015-3647-0

[CR138] Singh G et al (2025) Targeting EGFR and PI3K/mTOR pathways in glioblastoma: innovative therapeutic approaches. Med Oncol 42(4):97 40064710 10.1007/s12032-025-02652-1

[CR139] Sung H et al (2021) Global Cancer Statistics 2020: GLOBOCAN estimates of incidence and mortality worldwide for 36 cancers in 185 countries. CA Cancer J Clin 71(3):209–249 33538338 10.3322/caac.21660

[CR140] Tao S, Tao K, Cai X (2024) Pan-cancer analysis reveals PDK family as potential indicators related to prognosis and immune infiltration. Sci Rep 14(1):5665 38453992 10.1038/s41598-024-55455-1 PMC10920909

[CR141] Tariq K, Luikart BW (2021) Striking a balance: PIP(2) and PIP(3) signaling in neuronal health and disease. Explor Neuroprotective Ther 1:86–100 35098253 10.37349/ent.2021.00008 PMC8797975

[CR142] Tomuleasa C et al (2024) Therapeutic advances of targeting receptor tyrosine kinases in cancer. Signal Transduct Target Ther 9(1):201 39138146 10.1038/s41392-024-01899-w PMC11323831

[CR143] Toson B et al (2022) Targeting Akt/PKB in pediatric tumors: a review from preclinical to clinical trials. Pharmacol Res 183:106403 35987481 10.1016/j.phrs.2022.106403

[CR144] Trenker R, Jura N (2020) Receptor tyrosine kinase activation: from the ligand perspective. Curr Opin Cell Biol 63:174–185 32114309 10.1016/j.ceb.2020.01.016 PMC7813211

[CR145] Tsang JYS, Tse GM (2020) Molecular classification of breast cancer. Adv Anat Pathol 27(1):27–35 31045583 10.1097/PAP.0000000000000232

[CR146] Unni N, Arteaga CL (2019) Is dual mTORC1 and mTORC2 therapeutic blockade clinically feasible in cancer? JAMA Oncol 5(11):1564–1565 31465107 10.1001/jamaoncol.2019.2525

[CR147] Vadhan A et al (2022) CD44 promotes breast cancer metastasis through AKT-mediated downregulation of nuclear FOXA2. Biomedicines 10(10):2488 36289750 10.3390/biomedicines10102488 PMC9599046

[CR148] Varzideh F et al (2023) Molecular mechanisms underlying pluripotency and self-renewal of embryonic stem cells. Int J Mol Sci 24(9) 10.3390/ijms24098386 PMC10179698 37176093

[CR149] Vinogradova Y, Coupland C, Hippisley-Cox J (2020) Use of hormone replacement therapy and risk of breast cancer: nested case-control studies using the QResearch and CPRD databases. BMJ 371:m3873 33115755 10.1136/bmj.m3873 PMC7592147

[CR150] Wang N et al (2024) The landscape of PDK1 in breast cancer. Cancers (Basel) 14(3) 10.3390/cancers14030811 PMC8834120 35159078

[CR151] Wang Y, Minden A (2022) Current molecular combination therapies used for the treatment of breast cancer. Int J Mol Sci 23(19) 10.3390/ijms231911046 PMC9569555 36232349

[CR152] Wang D et al (2014) Oct-4 and Nanog promote the epithelial-mesenchymal transition of breast cancer stem cells and are associated with poor prognosis in breast cancer patients. Oncotarget 5(21):10803–10815 25301732 10.18632/oncotarget.2506 PMC4279411

[CR153] Wang XQ et al (2017) CDK1-PDK1-PI3K/Akt signaling pathway regulates embryonic and induced pluripotency. Cell Death Differ 24(1):38–48 27636107 10.1038/cdd.2016.84 PMC5260505

[CR154] Wang X et al (2019) *Body* mass index at diagnosis as a prognostic factor for early-stage invasive breast cancer after surgical resection. Oncol Res Treat 42(4):195–201 30852575 10.1159/000496548

[CR155] Wang Y et al (2022) Thioridazine combined with carboplatin results in synergistic inhibition of triple negative breast cancer by targeting cancer stem cells. Transl Oncol 26:101549 36191461 10.1016/j.tranon.2022.101549 PMC9530598

[CR156] Watt AC, Goel S (2022) Cellular mechanisms underlying response and resistance to CDK4/6 inhibitors in the treatment of hormone receptor-positive breast cancer. Breast Cancer Res 24(1):17 35248122 10.1186/s13058-022-01510-6 PMC8898415

[CR157] Wei Y et al (2022) ALDH1: a potential therapeutic target for cancer stem cells in solid tumors. Front Oncol 12:1026278 36387165 10.3389/fonc.2022.1026278 PMC9650078

[CR158] Wu X et al (2022) Beyond controlling cell size: functional analyses of S6K in tumorigenesis. Cell Death Dis 13(7):646 35879299 10.1038/s41419-022-05081-4 PMC9314331

[CR159] Wu B et al (2023) Cross-talk between cancer stem cells and immune cells: potential therapeutic targets in the tumor immune microenvironment. Mol Cancer 22(1):38 36810098 10.1186/s12943-023-01748-4 PMC9942413

[CR160] Wylaz M et al (2023) Exploring the role of PI3K/AKT/mTOR inhibitors in hormone-related cancers: a focus on breast and prostate cancer. Biomed Pharmacother 168:115676 37832401 10.1016/j.biopha.2023.115676

[CR161] Xia P, Xu XY (2015) PI3K/Akt/mTOR signaling pathway in cancer stem cells: from basic research to clinical application. Am J Cancer Res 5(5):1602–1609 26175931 PMC4497429

[CR162] Xiang X et al (2025) 3-Phosphoinositide*-*dependent kinas*e *1 as a therapeutic target for treating diabetes. Curr Diabetes Rev 21(4):47–56 38468518 10.2174/0115733998278669240226061329

[CR163] Xu H et al (2016) CD44 correlates with clinicopathological characteristics and is upregulated by EGFR in breast cancer. Int J Oncol 49(4):1343–1350 27499099 10.3892/ijo.2016.3639 PMC5021250

[CR164] Yamashina T et al (2014) Cancer stem-like cells derived from chemoresistant tumors have a unique capacity to prime tumorigenic myeloid cells. Cancer Res 74(10):2698–2709 24638980 10.1158/0008-5472.CAN-13-2169

[CR165] Yang F et al (2022) Correlation between androgen receptor expression in luminal B (HER-2 negative) breast cancer and disease outcomes. J Pers Med 12(12) 10.3390/jpm12121988 PMC9785183 36556209

[CR166] Yang L et al (2015) *LGR5* promotes breast cancer progression and maintains stem-like cells through activation of Wnt/beta*-*catenin signaling. Stem Cells 33(10):2913–2924 26086949 10.1002/stem.2083

[CR167] Yang L et al (2020) Targeting cancer stem cell pathways for cancer therapy. Signal Transduct Target Ther 5(1):1–35 32296030 10.1038/s41392-020-0110-5 PMC7005297

[CR168] Yedjou CG et al (2019) Health and racial disparity in breast cancer. Adv Exp Med Biol 1152:31–49 31456178 10.1007/978-3-030-20301-6_3 PMC6941147

[CR169] Yi K et al (2025) Bisphenol S exposure promotes stemness of triple-negative breast cancer cells via regulating Gli1-mediated Sonic hedgehog pathway. Environ Res 264(Pt 1):120293 39505130 10.1016/j.envres.2024.120293

[CR170] Yousefnia S et al (2020) Mechanistic pathways of malignancy in breast cancer stem cells. Front Oncol 10:452 32426267 10.3389/fonc.2020.00452 PMC7212408

[CR171] Yu F et al (2016) The combination of NVP-BKM120 with trastuzumab or RAD001 synergistically inhibits the growth of breast cancer stem cells in vivo. Oncol Rep 36(1):356–364 27175939 10.3892/or.2016.4799

[CR172] Yuan J et al (2024) Decoding tumor microenvironment: EMT modulation in breast cancer metastasis and therapeutic resistance, and implications of novel immune checkpoint blockers. Biomed Pharmacother 181:117714 39615165 10.1016/j.biopha.2024.117714

[CR173] Zeng X et al (2024) Targeting autophagy to enhance chemotherapy and immunotherapy in oral cancer. Front Immunol 15:1535649 39840028 10.3389/fimmu.2024.1535649 PMC11747659

[CR174] Zhang Y et al (2018) Epigenetic silencing of RNF144A expression in breast cancer cells through promoter hypermethylation and MBD4. Cancer Med 7(4):1317–1325 29473320 10.1002/cam4.1324 PMC5911569

[CR175] Zhang J et al (2025) Therapeutic targets in the Wnt signaling pathway: treating cancer with specificity. Biochem Pharmacol 236:116848 40049295 10.1016/j.bcp.2025.116848

[CR176] Zhao Z et al (2024) Wnt/beta-Catenin signaling pathway in hepatocellular carcinoma: pathogenic role and therapeutic target. Front Oncol 14:1367364 38634048 10.3389/fonc.2024.1367364 PMC11022604

[CR177] Zheng N et al (2023) Master kinase PDK1 in tumorigenesis. Biochim Biophys Acta Rev Cancer 1878(6):188971 37640147 10.1016/j.bbcan.2023.188971

[CR178] Zhou H et al (2019) B591, a novel specific pan-PI3K inhibitor, preferentially targets cancer stem cells. Oncogene 38(18):3371–3386 30635656 10.1038/s41388-018-0674-5 PMC6756013

